# Context Differences Reveal Insulator and Activator Functions of a Su(Hw) Binding Region

**DOI:** 10.1371/journal.pgen.1000159

**Published:** 2008-08-15

**Authors:** Alexey A. Soshnev, Xingguo Li, Misty D. Wehling, Pamela K. Geyer

**Affiliations:** 1 Program in Molecular and Cellular Biology, University of Iowa, Iowa City, Iowa, United States of America; 2 Department of Biochemistry, University of Iowa, Iowa City, Iowa, United States of America; European Molecular Biology Laboratory, Germany

## Abstract

Insulators are DNA elements that divide chromosomes into independent transcriptional domains. The Drosophila genome contains hundreds of binding sites for the Suppressor of Hairy-wing [Su(Hw)] insulator protein, corresponding to locations of the retroviral *gypsy* insulator and non-*gypsy* binding regions (BRs). The first non-*gypsy* BR identified, 1A-2, resides in cytological region *1A*. Using a quantitative transgene system, we show that 1A-2 is a composite insulator containing enhancer blocking and facilitator elements. We discovered that 1A-2 separates the *yellow* (*y*) gene from a previously unannotated, non-coding RNA gene, named *yar* for *y-achaete* (*ac*) intergenic RNA. The role of 1A-2 was elucidated using homologous recombination to excise these sequences from the natural location, representing the first deletion of any Su(Hw) BR in the genome. Loss of 1A-2 reduced *yar* RNA accumulation, without affecting mRNA levels from the neighboring *y* and *ac* genes. These data indicate that within the *1A* region, 1A-2 acts an activator of *yar* transcription. Taken together, these studies reveal that the properties of 1A-2 are context-dependent, as this element has both insulator and enhancer activities. These findings imply that the function of non-*gypsy* Su(Hw) BRs depends on the genomic environment, predicting that Su(Hw) BRs represent a diverse collection of genomic regulatory elements.

## Author Summary

Insulators are conserved genomic elements that define domains of independent transcription. One class of insulators in the Drosophila genome are defined by the binding of the Su(Hw) protein, with the *gypsy* insulator representing the classic Su(Hw)-dependent insulator. Su(Hw) associates with hundreds of non-*gypsy* regions distributed throughout the genome that differ in sequence and organization from the *gypsy* insulator. To gain insights into the role of Su(Hw) in genome organization, we defined the properties of the first non-*gypsy* Su(Hw) binding region identified, 1A-2. Our studies reveal differences in 1A-2 activity, depending on the context tested. We show that 1A-2 is an insulator in enhancer blocking studies but functions as a transcriptional activator within the natural genomic location. Our findings are reminiscent of properties of binding regions that associate with the vertebrate CTCF protein, which have defined insulator, activator, and repressor functions. Finally, our studies indicate that a noncoding RNA gene may contribute to independent transcriptional regulation in the genome.

## Introduction

In eukaryotic genomes, neighboring genes often display distinct spatial and temporal patterns of transcription, even though intergenic distances are within the range of enhancer and silencer action. These observations suggest that constraints exist that limit promiscuous interactions between long distance regulatory elements and non-target promoters. Chromatin insulators represent one class of genomic elements that restrict enhancer and silencer action [1]–[5].

Insulators have been identified based on two functional properties. First, insulators prevent enhancer and silencer modulation of a promoter in a position-dependent manner, such that an enhancer or silencer is blocked only when the insulator is located between these elements and a promoter. Second, insulators protect gene expression from positive and negative chromosomal position effects associated with ectopic placement of genes within genomes, an activity referred to as barrier function. Sequences with one or both of these properties have been identified in most eukaryotic genomes and have been implicated in the regulation of diverse cellular processes, ranging from centromere function in yeast to imprinting in mammals [6],[7]. These observations imply that insulators are fundamental components of eukaryotic genomes.

One of the best-characterized insulators resides in the 5′ untranslated region of the Drosophila *gypsy* retrovirus. This versatile *gypsy* insulator blocks over twenty enhancers active in different tissues and developmental stages, prevents repressive effects caused by Polycomb group complexes and heterochromatin and protects an origin of DNA replication from chromosomal position effects [2],[5]. The *gypsy* insulator consists of a cluster of twelve repeats that are bound by the zinc finger Suppressor of Hairy-wing [Su(Hw)] protein [8]. At least three additional proteins are associated with the *gypsy* insulator, including Modifier of (mdg4) 67.2 (Mod67.2), Centrosomal Protein of 190 kD (CP190) and Enhancer of *y^2^* [E(y)2]. In general, Mod67.2 and CP190 are required for enhancer and silencer blocking by the *gypsy* insulator, while E(y)2 has been shown to be required only for barrier function [9]–[13].

The Su(Hw) protein associates with hundreds of non-*gypsy* regions in the Drosophila genome that have a largely unknown function. The extensive co-localization of the four *gypsy* insulator proteins at non-*gypsy* regions has led to the proposal that these represent chromatin insulators. Yet, non-*gypsy* Su(Hw) binding regions are different in sequence and organization from the *gypsy* insulator, with the majority of BRs containing single Su(Hw) binding sites (BSs) [14]–[18]. This observation is striking, as at least four tightly spaced Su(Hw) sites from the *gypsy* insulator were required for robust enhancer blocking [19]–[21]. Direct tests of the non-*gypsy* BRs in transgene assays show that most, but not all, interfere with enhancer-activated transcription [15]–[18]. These findings imply that non-*gypsy* regions contain additional elements that assist the insulator function of Su(Hw).

**Figure 1:**
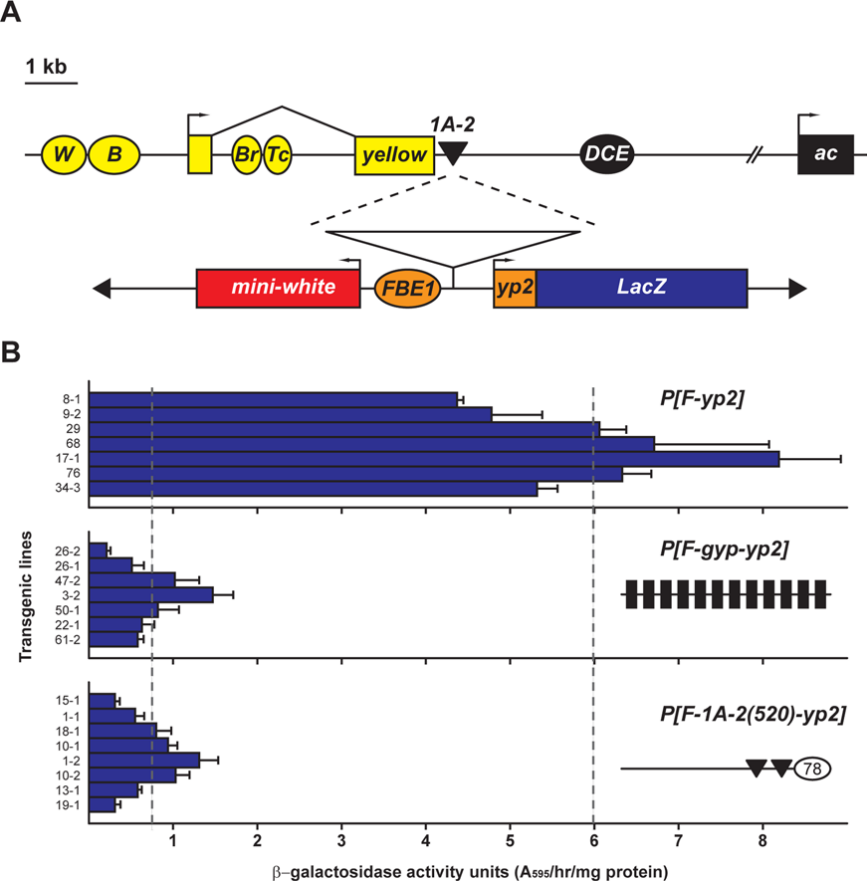
The 1A-2 insulator. A. Top: The cuticle pigmentation *yellow (y)* gene contains two exons (yellow rectangles) and four tissue specific enhancers (ovals marked W for wing, B for body, Br for bristle and Tc for tarsal claw). The proneural *achaete (ac)* gene contains one exon (black rectangle). The tissue-specific enhancer in the upstream regulatory region is shown (oval marked *DCE* for dorsocentral enhancer). 1A-2 Su(Hw) BR is located downstream of the *y* gene, separating this gene from the *ac* regulatory region. Bottom: Structure of the *FBE1-yp2-LacZ* transgene used to define the sequences of 1A-2 required for enhancer blocking. Subregions of 1A-2 were inserted between the *FBE1* enhancer and *yp2* promoter. Effects of transcriptional activation were determined through enzymatic assay that tested β-galactosidase activity. The mini-*white (w)* gene was used for identification of transgenic flies in germ line transformation. B. β-galactosidase activity associated with transgenic lines carrying transposons derived from *FBE1-yp2-LacZ*. Each bar represents the average activity units (aau) for independent insertion lines corresponding to the indicated transposon (right). When pertinent, a cartoon is shown that represents the structure of sequences included in the *FBE1-yp2-LacZ* transgene. The *gypsy* Su(Hw) BS are shown as black rectangles, the 1A-2 Su(Hw) BS are shown as black triangles, a novel 1A-2 element is shown as an oval carrying 78 bp. Assays were completed on extracts isolated from females representing at least three independent crosses. Error bars indicate standard deviation. The vertical dashed line on the left represents the aau value for flies carrying *P[F-gyp-yp2]*, while the vertical dashed line on the right represents the aau value for flies carrying *P[F-yp2]*.

The first non-*gypsy* Su(Hw) BR identified, named 1A-2, is a cluster of two Su(Hw) BSs located in cytological region *1A* (Figure 1). Here we investigated the properties of 1A-2, using two strategies. First, we employed a quantitative transgene system to define the 1A-2 sequences required for enhancer blocking. Second, we performed homologous recombination to establish lines carrying a deletion of 1A-2 at the natural genomic location, representing the first deletion of a non-*gypsy* Su(Hw) BR in the Drosophila genome. Effects of the loss of these sequences on gene expression in the *1A* region were determined, leading to the discovery that 1A-2 contributes to transcriptional activation of a novel, non-coding RNA gene. Taken together, our studies demonstrate that 1A-2 has both activator and insulators properties, depending on the context tested. These findings imply that properties of non-*gypsy* Su(Hw) BRs are influenced by the genomic environment, predicting that Su(Hw) BRs represent a diverse collection of elements with distinct regulatory functions.

## Results

### 1A-2 Is a Composite Insulator

The Su(Hw) BR 1A-2 is a 520 bp element that contains two Su(Hw) BSs [18] and a CP190 BS [9]. Previous studies using qualitative transgene assays demonstrated that 1A-2 blocked enhancer-activated transcription in a position-dependent manner, a key feature of insulator activity [17],[18]. We employed the quantitative *Fat Body Enhancer (FBE)1*-*yolk protein (yp)2* -*LacZ* transgene to define DNA sequences required for 1A-2 enhancer blocking (Figure 1), a system previously used to characterize properties of the *gypsy* insulator [20],[22]. A reporter transgene was constructed wherein full length 1A-2(520) was inserted between *FBE1* and the *yp2* promoter. Multiple *P[F-1A-2(520)-yp2]* transgenic lines with single insertions were established. Quantitative β-galactosidase activity assays were completed to define the level of *yp2* promoter activity. Protein extracts were isolated from adult females representing several independent lines, and multiple assays were undertaken to establish an average activity unit (aau) for each transgene (Figure 1). We found that transgenic *P[F-1A-2(520)-yp2]* females had low levels of *yp2* expression (aau 0.86), similar to levels in *P[F-gyp-yp2]* females (aau 0.75) and significantly lower than levels found in the control *P[FBE1-yp2]* females (aau 5.97). We conclude that 1A-2 blocks *FBE1*, extending the enhancer blocking effects of 1A-2 to a new enhancer-promoter pair.

**Figure 2:**
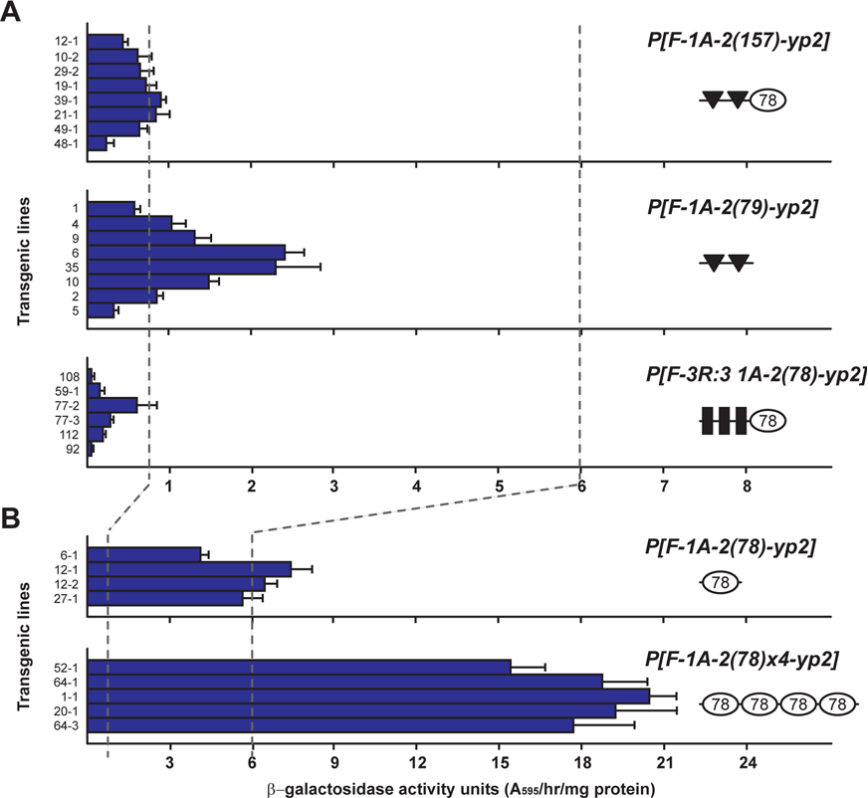
1A-2 contains enhancer blocking and facilitator elements. (A,B) β-galactosidase activity of independent transgenic lines carrying a derivative of the *FBE1-yp2-LacZ* transgene. The name and structure of the insertion is shown on the right, including the 3′ 157 bp 1A-2 fragment (*P[F-1A-2(157)-yp2]*), the 79 bp fragment with only two Su(Hw) BSs (*P[F-1A-2(79)-yp2]*), the 3′ 78 bp fragment combined with a Su(Hw) BR containing three copies of site 3 from the *gypsy* insulator (*P[F-3R:3 1A-2(78)-yp2]*), the 1A-2 78 bp fragment (*P[F-1A-2(78)-yp2]*) and four copies of the 3′ 1A-2 78 bp fragment (*P[F-1A-2(78)×4-yp2]*). Error bars indicate the standard deviation (n = 3). The vertical dashed line on the left represents the aau value for flies carrying *P[F-gyp-yp2]*, while the vertical dashed line on the right represents the aau value for flies carrying *P[F-yp2]*. In B, the X-axis scale is increased three fold. Symbols are as described in Figure 1.

The minimal sequences required for 1A-2 insulator function were determined by generation of transgenic lines carrying transposons with insertion of subregions of 1A-2 between *FBE1* and *yp2-LacZ* (Figure 2). *P[F-1A-2(157)-yp2]* females showed a strong enhancer block (aau 0.62). As this subregion lacks the CP190 BS [9], these findings indicate that direct CP190 binding is not required for insulator function. 1A-2(157) was further divided into two parts, one containing the two Su(Hw) BSs, 1A-2(79), and one containing the remaining sequences, 1A-2(78). Transgenic *P[F-1A-2(79)-yp2]* females showed a two-fold weaker enhancer block than 1A-2(157) (aau 1.29, P = 0.02), whereas *P[F-1A-2(78)-yp2]* females showed high *yp2* activity levels, close to those obtained for the control *P[F-yp2]* females (aau 5.9 versus 5.97). These data suggest that 1A-2(78) contributes to the blocking effectiveness of the 1A-2 Su(Hw) BSs, but cannot itself block enhancer-promoter interactions.

We considered two possibilities to account for the contributions made by 1A-2(78). First, these sequences might contain a binding site(s) for a second insulator protein that cooperates with the Su(Hw) BSs for insulator function. Second, 1A-2(78) might improve the activity of the Su(Hw) BSs, perhaps by increasing *in vivo* association. We reasoned that if 1A-2(78) contained a binding site for a novel insulator protein, then insulator effects might require a reiterated element, as observed previously when individual binding sites for other insulator proteins were tested [23],[24]. To this end, we generated *P[F-1A-2 (78×4)-yp2]* that carried four copies of 1A-2(78) inserted between *FBE1* and the *yp2* promoter. Surprisingly, these transgenic females had higher *yp2* activity than the control *P[F-yp2]* females (aau 18.78 versus 5.97 aau, P = 6.3×10^−8^). Transgenic *P[F-1A-2(78×4)-yp2]* males showed no *yp2* activity (data not shown). Based on the retained transcriptional specificity of the *P[F-1A-2 (78×4)-yp2]* transgene, we conclude that 1A-2(78) is not a general transcriptional enhancer but improves the activity of FBE1. These data imply that 1A-2(78) may possess a general activity that facilitates factor association. To test this postulate, we determined whether 1A-2(78) restored enhancer blocking to a synthetic Su(Hw) BR containing three reiterated *gypsy* BSs (3R:3) that was previously shown to be inactive in this transgene system [20]. Supporting a facilitator function of 1A-2(78) we found that transgenic *P[F- 3R:3-1A-2(78)-yp2]* females had low *yp2* activity (aau 0.22). These studies show that in the presence of 1A-2(78), 3R:3 became a strong insulator. As previous findings suggest that the effectiveness of enhancer blocking by the Su(Hw) protein is limited by the *in vivo* accessibility of Su(Hw), we conclude 1A-2(78) is a facilitator that may improve transcription factor binding to chromosomes.

### The *y-ac* Intergenic Region Contains a Novel, Non-Coding RNA Gene

**Figure 3:**
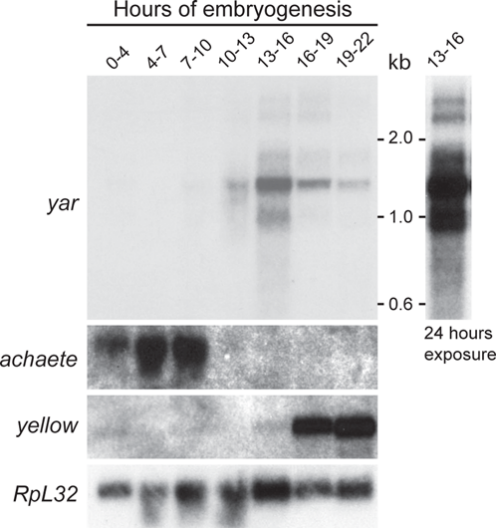
Transcription of *yar* is regulated during embryogenesis. Northern analysis of five µg of polyA^+^ RNA isolated from staged embryo collections. The blot was probed with a *yar* cDNA, and two exposures are shown: 3 hours (left) and 24 hours (right). The blot was stripped and reprobed with DNA sequences corresponding to *y*, *ac* and the constitutively active *RpL32* gene that served as a loading control.

As a first step in defining the role of 1A-2 within the *y-ac* region, we evaluated whether the existing annotation reflected the transcriptional potential of this region. These analyses were motivated by the recent studies showing widespread transcription in intergenic regions of the Drosophila genome [25]. A search of the NCBI databases uncovered a small, novel, processed EST of ∼400 nt that was transcribed from the *y-ac* intergenic sequences. Sequences corresponding to this EST are located ∼1.4 kb downstream of the *y* termination signal and transcribed in the same direction as the *y* and *ac* genes. Northern analyses of embryonic polyA^+^ RNA using a radiolabeled probe representing the intergenic EST identified a family of RNAs, with the most abundant species sized at ∼1.6 kb (Figure 3). Accumulation of these RNAs began ∼7 hours after the start of embryogenesis, in agreement with the expression profile obtained using tiling array studies of embryonic RNAs [25]. These data suggest that the *y-ac* intergenic region contains a previously uncharacterized gene, which we call *yar*, for *y-ac intergenic RNA*. Activation of genes in the *1A* locus is temporally in an order following chromosomal position, such that *ac*, then *yar* and then *y* is transcribed.

**Figure 4:**
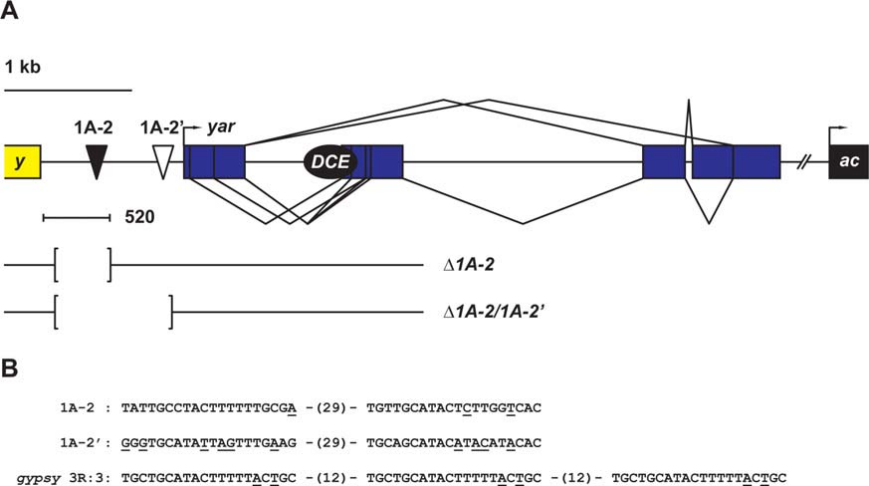
Detailed structure of control elements in the region separating the *y* and *ac* genes. A. The region separating the *y* and *ac* genes contains a cluster of two strong Su(Hw) BS (black triangle, 1A-2) and two weak Su(Hw) BS (white triangle, 1A-2′). 1A-2′ is located 85 bp upstream of the most upstream *yar* transcription start site (bent arrow). The alternative splicing pattern is shown: thin lines represent introns and blue rectangles indicate exons. The *ac* DCE (black oval) resides within *yar*. The limits of the original 520 bp 1A-2 insulator are shown as a bracketed line marked 520. The extent of the regions deleted in the *y^Δ1A-2^* (Δ1A-2) and *y^Δ1A-2/Δ1A-2′^* (Δ1A-2/1A-2′) flies are shown, where the bracketed regions were removed. B. The sequence of the Su(Hw) BSs in 1A-2 (top), 1A-2′ (middle), and the synthetic *gypsy* insulator (3R:3) (bottom), with the numbers indicating the distance of separation between BSs. The nucleotides different from the genomic Su(Hw) consensus BS [14] are underlined.

The structure of the *yar* RNAs was defined using rapid amplification of cDNA ends (RACE, Figure 4). Sequence analysis of the 5′ RACE products revealed three discrete transcription start sites within an ∼200 bp region, with the most distal RNA starting ∼1.2 kb downstream of the *y* gene. Each putative start site showed weak homology to Drosophila transcriptional control elements [26], with two having a partial match to the TATA consensus sequence located 17 to 35 bp upstream of the start site. Sequence analysis of the 3′ RACE products identified multiple splice variants, each ending in a common exon that contained an unconventional polyadenylation signal sequence AAATACA, previously estimated to be present in ∼3% of Drosophila genes [27], that was located 12 bp upstream of the string of As in the RACE products. Predicted translation of the *yar* RNAs indicated that no transcript would encode a protein of more than 75 amino acids, implying that *yar* is a non-coding RNA gene.

### Loss of 1A-2 Does Not Alter Adult Phenotypes Generated by *y* and *ac* Expression

**Figure 5:**
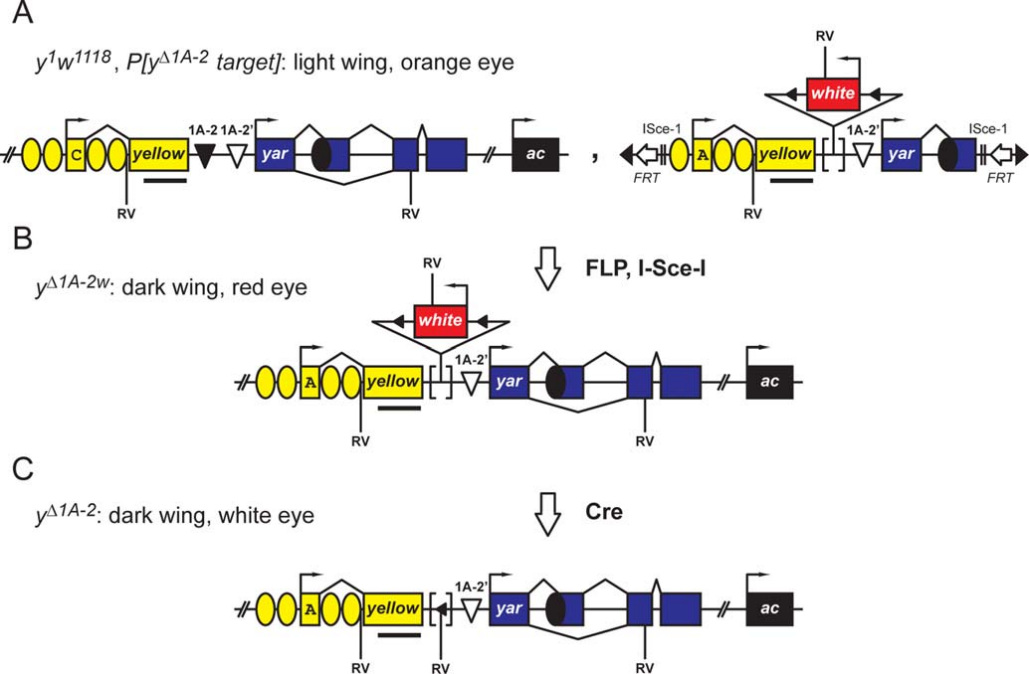
Ends-out targeting strategy to generate deletions of 1A-2 at the endogenous *1A* locus. A. Transgenic flies were generated that carried the mutant *y^1^* allele (structure shown where C indicates the mutation of the translation ATG start) at the endogenous *X* chromosome location (left) and the *P[y^Δ1A-2^ target]* transposon on a different chromosome (right) that carries a *y* gene lacking the wing enhancer, but encodes a wild type RNA (A indicates the presence of the correct translation ATG start). In this transposon, the *y* gene, flanked by FRT sites (white arrows) and *I-Sce*I sites, is within a *P* transposon (inverted black triangles). Other symbols representing the *y*, *yar* and *ac* genes are as described in Figure 1. Transgenic flies *y^1^ w^1118^*, *P[y^Δ1A-2^ target]* had a light wing color and orange eyes. B. FLP and *I-Sce*I enzymes catalyzed replacement of the *y^1^* allele at the endogenous locus, with *y^Δ1A-2w^*, in which the 1A-2 insulator is substituted by the *w^hs^* gene (raised triangle) inserted between *loxP* sites (black arrowheads on raised triangle). The recombinant *y^Δ1A-2w^* flies had dark wings and red eyes. C. Cre recombinase deleted the *w^hs^* gene, leaving behind a single *loxP* site to form *y^Δ1A-2^*. In the case of the *y^Δ1A-2^*, the remaining *loxP* site was mutated, forming a new *Eco*RV site (RV), whereas in the similarly derived *y^Δ1A-2/Δ1-A2′^* flies a wild type *loxP* site remained. The bar under the *y* gene indicates the probe used in the Southern analyses (see Figure S1).

Ends out gene targeting was used to delete 1A-2 from the *y-ac* region (Figures 4, 5). Gene targeting is a two step processes that requires establishment of transgenic flies that carry a transposon with the replacement gene, followed by the introduction of endonucleases to stimulate homologous recombination between the replacement gene and its endogenous homologue. To delete 1A-2, we constructed *P[y^Δ1A-2^ target]*. This transposon carried a modified *y* gene, wherein 1A-2 was replaced by the hypomorphic *w^hs^* gene that was flanked by *loxP* sites (Figure 5). Transgenic lines were established in a *y^1^ w^1118^* background, where the endogenous *y* gene carried a mutation of the translation start codon, and the endogenous *w* gene carried a deletion of the promoter. *P[y^Δ1A-2^ target]* flies had orange eyes and dark pigmentation of all cuticle structures except the wing, as the *y* gene lacked the wing enhancer. To stimulate recombination, transgenic *y^1^ w^1118^*; *P[y^Δ1A-2^ target]* males were crossed to females carrying the *heat shock (hs)-FLP* recombinase and the *hs*-*I-Sce*I endonuclease transgenes and progeny of this cross were heat shocked to produce the endonucleases. Over 100 resulting females were crossed to *y^1^ w^1118^* males and homologous recombinants were identified among the offspring of this cross in two ways. First, flies were screened for dark wings, as recombination at the endogenous *y^1^* gene would reconstitute a wild type *y* transcription unit with all enhancers, whereas progeny with ectopic insertions of the replacement *y* gene would produce flies with lightly colored wings due to the absent wing enhancer. Second, we conducted genetic analyses to determine whether the *w^+^* phenotype was linked to the *X* chromosome. Five putative homologous recombination lines were established based on dark wing pigmentation. Further genetic analyses showed that in one line, XGL339-23-38, the *w* marker mapped to the *X* chromosome, suggesting a correct targeting event. Southern analyses confirmed the structure of the *y* gene in these flies (Figure S1). This targeted allele was named, *y^Δ1A-2w^*.

We reasoned that if 1A-2 was an insulator in the *y-ac* locus, then deletion of 1A-2 would release constraints on the *y* and *ac* enhancers, causing changes in gene expression that would alter cuticle pigmentation and bristle number in *y^Δ1A-2w^* relative to wild type flies [28],[29]. However, we found that adult phenotypes of *y^Δ1A-2w^* flies were indistinguishable from wild type flies. In *y^Δ1A-2w^*, the *w^hs^* gene replaced 1A-2. To rule out the possibility that this gene served as a surrogate insulator by carrying a promoter that captured the *y* and *ac* enhancers, *y^Δ1A-2w^* flies were crossed to flies carrying a source of Cre recombinase to remove the *w^hs^* gene. Southern and PCR analyses confirmed the structure of *y* gene in *y^Δ1A-2^* flies (Figure S1). Again, the cuticle and bristle phenotypes of the *y^Δ1A-2^* flies were indistinguishable from wild type. Taken together, these data imply that 1A-2 is not an insulator at the endogenous genomic location.

Within the *y-ac* intergenic region, we identified a second cluster of Su(Hw) binding sites, which we called 1A-2′. These sites differ from the Su(Hw) consensus sequence at multiple highly conserved positions (Figure 4). Electrophoretic mobility shift assays demonstrated that 1A-2′ had ∼3-fold lower affinity for Su(Hw) than 1A-2 (data not shown). Even so, we considered it possible that weaker 1A-2′ Su(Hw) BR might provide a redundant function with 1A-2 to define regulatory interactions in the *y-ac* region. For this reason, we generated a second targeting vector, *P[y^Δ1A-2/Δ1A-2′^ target]*, wherein the *w^hs^* gene replaced an ∼1.0 kb deletion that encompassed both 1A-2 and 1A-2′. Following the procedure described above, six putative homologous recombinant lines were identified based on dark wing pigmentation. Further genetic analyses showed that one of these lines, XGL426-41-4, had marker linkage to the *X* chromosome. This allele was named *y^Δ1A-2/Δ1A-2′w^*. Flies from this line were used to obtain a derivative line lacking the *w^hs^* gene, producing *y^Δ1A-2/Δ1A-2′^*. Southern and PCR analyses confirmed the structure of the *y* gene resulting from these targeting events (Figure S1). Comparison of adult phenotypes in *y^Δ1A-2/Δ1A-2′^* and wild type flies showed that the cuticle color and bristle number were indistinguishable, suggesting that 1A-2′ did not compensate for 1A-2.

#### *yar* Expression Is Lowered in the Absence of 1A-2

**Figure 6:**
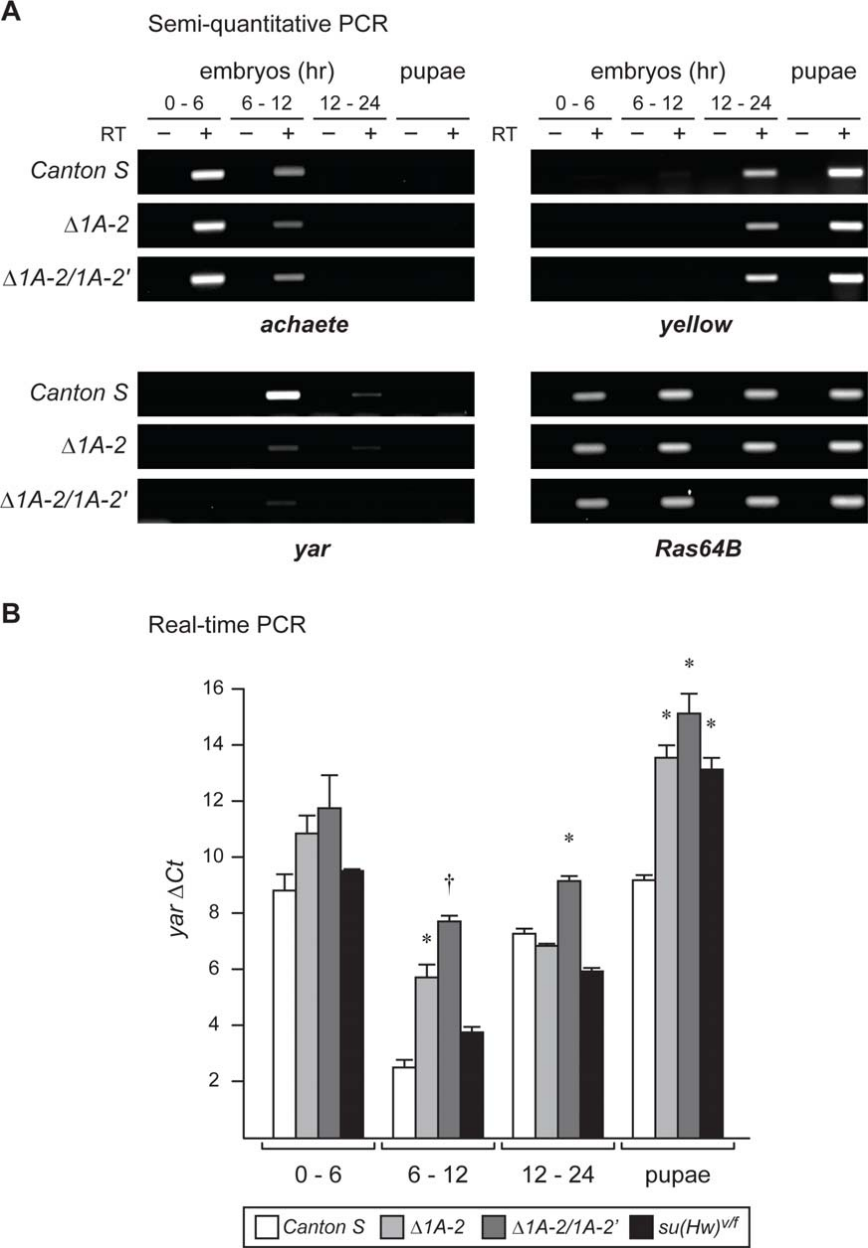
Loss of 1A-2 and 1A2′ reduces *yar* RNA accumulation during embryogeneis. A. Ethidium bromide stained PCR products obtained from semi-quantitative PCR to evaluate *ac*, *y*, *yar* and *Ras 64B* RNA levels in wild type (Canton S), *y^Δ1A-2^* (Δ1A-2) and *y^Δ1A-2/Δ1A-2′^* (Δ1A-2/Δ1A-2′). *Ras64B* is constitutively expressed and serves as a control. The minus (−) RT lanes control for genomic DNA contamination. Different stages of embryonic and mixed pupal RNA were analyzed. B. Quantitative real time PCR (Q-PCR) was used to determine levels of *yar* mRNA accumulation from RNAs isolated during development from wild type and mutant lines. Individual transcript levels defined by Q-PCR were normalized to *Ras64B* for amount of input cDNA (ΔC_T_). A larger ΔC_T_ indicates a reduction in RNA. Error bars indicate standard deviation of values obtained from analyses of three independently isolated RNAs. Significant changes in RNA accumulation relative to wild type are as indicated (*, P = <0.01; †, P<0.001, Student's two-tailed t-test).

We postulated that changes in *y* and/or *ac* gene expression might occur, but that these differences may not be readily observed in analyses of adult phenotypes. For this reason, levels of RNA accumulation were quantified using reverse transcriptase PCR (Figure 6). Total RNA was isolated from staged collections of Canton S (wild type), *y^Δ1A-2^* and *y^Δ1A-2/Δ1A-2′^* embryos and pupae, representing the developmental periods where the *y* and *ac* genes are maximally expressed [30]. Following conversion to cDNA, templates were amplified using primers against *y*, *ac*, *yar*, and *Ras64B*, a constitutively expressed RNA [16],[31]. In a first set of experiments, PCR products obtained from cDNA amplification in the linear range were run on an agarose gel and visualized by ethidium bromide staining (Figure 6A semi-quantitative, Figure S2). These studies revealed that the loss of 1A-2 and 1A-2′ did not change the timing or level of *y* and *ac* RNA accumulation, consistent with the lack of phenotypic changes. In contrast, amplification of *y^Δ1A-2^* and *y^Δ1A-2/Δ1A-2′^* cDNA showed reduced *yar* levels relative to Canton S, without a temporal change. These findings indicate that 1A-2 and 1A-2′ are required for *yar* expression.

Quantitative real time PCR analyses (Q-PCR) were undertaken to test the semi-quantitative results (Figure 6B, Figure S3). In these studies, we included analysis of *scute* (*sc*) RNA accumulation, the gene downstream of *ac*. A cycle threshold (C_T_) for each primer set was determined and a corresponding ΔC_T_ was calculated, using the *Ras64B* C_T_ for standardization. These analyses identified a significant increase in ΔC_T_ for *yar* within *y^Δ1A-2^* and *y^Δ1A-2/Δ1A-2′^* samples, relative to Canton S. These data correspond to a 7- and 25-fold decrease in *y^Δ1A-2^* embryonic and pupal *yar* RNA respectively and a 32- and 41-fold decrease in *y^Δ1A-2/Δ1A-2′^* embryonic and pupal *yar* RNA (Figure 6B, Figure S3). These data suggest that within the context of the *y-ac* genomic region, 1A-2 and 1A-2′ serve as an enhancer of the non-coding *yar* gene.

To determine whether the Su(Hw) contributes to expression of genes in the *1A* region, we quantified of *y*, *yar*, *ac* and *sc* RNAs in a *su(Hw)^v^/su(Hw)^f^* mutant background, using Q-PCR. The *su(Hw)^v^* allele carries a promoter deletion and the *su(Hw)^f^* allele carries a point mutation that produces a full-length protein with an inactivate finger 10 [32]. We found that only the level of pupal *yar* RNA was significantly changed in the *su(Hw)^v/f^* mutant background, associated with an ∼21-fold decrease (Figure 6B, Figure S3). These data indicate that Su(Hw) makes positive contribution to the normal low level of pupal *yar* transcription. The absence of expression changes in embryonic RNA may be confounded by an ability of Su(Hw)^f^ to bind to 1A-2 in early embryos. Previous studies have shown that disruption of Su(Hw) zinc finger 10 limits chromosome accessibility, without altering DNA recognition [32]. It is possible that in early embryos, 1A-2 is in a more accessible chromatin structure, thereby allowing Su(Hw)^f^ to bind 1A-2 and activate *yar*, but that this property is lost during development. We are unable to test *yar* expression in a *su(Hw)* null background, as complete loss of Su(Hw) blocks oogenesis. Regardless, our data imply that Su(Hw), along with contributions made by other proteins associated with 1A-2, function as an activator of *yar* transcription during development.

## Discussion

Prevailing models of *gypsy* insulator function predict that the *gypsy* insulator establishes independent transcriptional domains through cooperation with genomic insulators defined by non-*gypsy* Su(Hw) BRs. Recent findings indicate that the sequence and organization of non-*gypsy* BSs differ from the Su(Hw) BR in the *gypsy* retrovirus [14]–[16]. These observations imply that properties of non-*gypsy* BRs may be distinct from those of the *gypsy* insulator. We defined the properties of 1A-2, to gain insights into mechanisms of Su(Hw) insulator action.

### Enhancer Blocking by 1A-2 Requires Su(Hw) BSs and a Facilitator

We used the quantitative *FBE1-yp2-LacZ* reporter system to define the sequence requirements for enhancer blocking by 1A-2(520). Prior application of this system demonstrated that at least four *gypsy* Su(Hw) sites were needed for robust blocking [20]. Here, we show that 1A-2(157) provided as strong an enhancer block as the *gypsy* insulator (Figures 1, 2). A fragment containing only the Su(Hw) BRs [1A-2(79)] reconstituted a weaker enhancer block than 1A-2(157), but had a greater blocking capacity than the synthetic insulators made from reiterated copies of BS3 of the *gypsy* insulator [20]. While we do not know the reason for the more robust blocking, we note that these regions differ in sequence and distance of separation from Su(Hw) sites (Figure 4). Blocking effectiveness does not appear to be due to differences in DNA recognition, as the *in vitro* binding constants for Su(Hw) for the 1A-2 and *gypsy* BSs are similar [16]. Our experiments revealed that 1A-2 contains a second regulatory element located in 1A-2(78). When these sequences were positioned next to the inactive, synthetic Su(Hw) BR (3R:3), a functional insulator was reconstituted (Figure 2B). These data are consistent with previous findings that Su(Hw) chromosome association is limited [32]. Taken together, we propose that 1A-2 is a composite insulator that contains an enhancer blocking and a facilitator function that may improve Su(Hw) chromosome association. Further, we predict that *in vivo* effectiveness of enhancer blocking by the Su(Hw) protein is related to the accessibility of Su(Hw) BSs. If single or small clusters of Su(Hw) BSs are located in genomic regions of open chromatin, then these regions will demonstrate enhancer blocking, as defined in transgene assays. This proposal implies that genomic context greatly influences the properties of non-*gypsy* Su(Hw) BRs.

### A Novel Non-Coding RNA Gene Separates the *y* and *ac* Genes in the *1A* Locus

1A-2 is located between the independently regulated *y* and *ac* genes. Chromatin immunoprecipitation studies demonstrated that 1A-2 is associated with Su(Hw), Mod67.2 and E(y)2 *in vivo* [12],[16],[18], suggesting that this element binds a complex competent for establishing a genomic insulator. Based on these properties, we postulated that 1A-2 was responsible for the regulatory independence of the *y* and *ac* genes in the *1A* locus [16]. As a first step in testing this proposal, we investigated transcription in the *y-ac* region to evaluate the current accuracy of the genomic annotation of this region. These studies identified a previously unannotated gene, *yar*, located ∼1.2 kb downstream of the *y* gene and ∼3.0 kb upstream of *ac*. Multiple, differentially spliced, polyA^+^ RNAs are encoded by *yar*, with the largest translation product predicted to be 75 amino acids, indicating that this is a non-coding RNA gene. Emerging data suggest that non-coding RNAs are abundant in eukaryotes and have a wide repertoire of biological functions, ranging from structural components in protein complexes to regulatory molecules involved in transcription and translation [33]–[35]. It is unknown whether *yar* has a function. As flies carrying a large genomic deletion that removes sequences upstream of *y* and extends downstream of *ac* (*y^−^ ac^−^*) are viable and fertile, *yar* is a non-essential gene.

### 1A-2 Is Required for Expression of a Non-Coding RNA Gene

Having re-defined the transcriptional profile in the *1A* locus, we tested the function of 1A-2 and a second, weaker Su(Hw) BR, 1A-2′, on gene regulation, using gene targeting to delete these elements. Our studies represent the first deletional analysis of any non-*gypsy* Su(Hw) BR in the Drosophila genome. Two targeted deletion lines, *y^Δ1A-2^* and *y^Δ1A-2/Δ1A-2′^* were established (Figure 4). Levels of *y*, *ac*, *sc* and *yar* RNA accumulation during development were studied using quantitative PCR. We find that loss of 1A-2 and 1A2′ has no effect on the timing and level of *y*, *ac* or *sc* RNAs relative to the wild type control (Figure S3), but strongly reduced *yar* RNA (Figure 6). These data suggest that the effects of loss of 1A-2 are limited to local changes of gene expression, implying that these sequences are not a chromatin insulator at the endogenous location. Instead, our data indicate that 1A-2 may be an activator of *yar* expression, consistent with other studies that have suggested a role for Su(Hw) in gene activation [36]–[38]. These data, coupled with genetic studies on the effects of the loss of Su(Hw) on expression of genes adjacent to Su(Hw) BRs [16], demonstrate that Su(Hw) BRs have diverse functions in the genome.

The complexity of the transcriptional effects associated with Su(Hw) BRs is reminiscent of regions in mammalian genomes that bind the versatile regulatory protein CTCF. High throughput genomic analyses have identified hundreds of CTCF binding sites within the mouse and human genomes [7], [39]–[41]. Although many of these sequences possess enhancer blocking activity [39],[42],[43], CTCF has been implicated in transcriptional activation [44]–[46], repression [47]–[50], and chromosome pairing [44],[51],[52]. These observations suggest that, similar to the non-*gypsy* Su(Hw) BRs, genomic context will have an important influence on the properties of CTCF BSs within a given region.

The mechanism(s) used to maintain transcriptional autonomy in the *1A* locus are unclear. The discovery of *yar* provides an alternative explanation to the need for a chromatin insulator. Based on the developmental timing displayed by the *1A* genes, we postulate that activation of *yar* transcription may cause inactivation of *ac* through transcriptional interference. Similarly, activation of *y* may repress *yar* transcription. Although *y^Δ1A-2^* and *y^Δ1A-2/Δ1A-2′^* flies show reduced *yar* expression, transcription is not abolished, suggesting that the remaining *yar* activity may be sufficient to turn off *ac*. Alternatively, other mechanisms can be considered that might influence enhancer preference, including selectivity of enhancers for certain classes of promoters [53],[54], the presence of promoter targeting sequences that direct enhancer action [55],[56], or promoter tethering elements that capture enhancers [57]. Further experiments to define the properties of DNA elements within the *1A* locus will resolve how transcriptional independence is achieved.

## Materials and Methods

### Fly Stocks and Crosses

Flies were raised at 25°C, 70% humidity on standard corn meal/agar medium. Description of the alleles used can be found at http://flybase.bio.indiana.edu.

### Construction of *FBE1-yp2* -*LacZ* Reporter Genes

The *FBE1-yp2* -*LacZ* fusion gene [20] carried a *Bgl*II site, positioned at −335 relative to the transcription start site (TSS) that was used for insertion of tested 1A-2 fragments. Resulting transgenes were inserted into a *P* element transformation vector, generating *P[F-1A-2(520)-yp2]* with the full length 1A-2, *P[F-1A-2(157)-yp2]* with a 157 bp region of 1A-2, *P[F-1A-2(79)-yp2]* with two 1A-2 Su(Hw) binding sites, *P[F-1A-2(78)-yp2]* with the 78 bp 3′ region, *P[F-1A-2(78×4)-yp2]* with four tandem repeats of the 1A-2 78 bp element and *P[F-3R:3(78)-yp2]* with a hybrid insertion between a cluster of three tandem repeats of the *gypsy* Su(Hw) binding sites [nucleotides 732–759 [58]], as described in [20] and the 78 bp element. *P* transposons were injected into the host *y^1^w^67c23^* strain or *w^1118^* (Genetic Services, Inc, Cambridge, MA). Transgenic lines were analyzed by Southern and PCR analyses to determine the number and integrity of the transposons. Lines with single transposon insertions were used in subsequent analyses.

### β-Galactosidase Spectrophotometric Assay

The *yp2* promoter activity was assessed using quantitative β-galactosidase assays, performed essentially as previously described [20]. Each transgenic line was assayed using extracts isolated from three different matings. Each extract was assayed in duplicate, and the error between these samples was less than 10%. Average promoter activity and standard deviation were determined using the statistical analysis feature of the Microsoft Excel program.

### Ends out Gene Targeting

Two targeting transposons were constructed for gene targeting, using *pW25* [59]–[61]. This vector has multi-cloning site, *Not*I-*Sph*I-*Acc*65I-Stop-*lox-w^hs^-lox*-Stop-*Asc*I-*Bsi*WI. The *lox* sites are in direct orientation, permitting removal of the *w^hs^* transformation marker by Cre recombinase. *P[y^Δ1A-2^ target]* (XGL339) was used to target an ∼0.43 kb deletion encompassing 1A-2 alone, whereas *P[y^Δ1A-2/Δ1A-2′^ target]* (XGL426) was used to target an ∼1.03 kb deletion that included 1A-2 and 1A-2′. These targeting transposons were generated in a two-step procedure. First, a 6.6 kb *yellow* fragment (−1842 to +4796 relative to the *y*TSS) was PCR amplified, using primers carrying the *Bsi*WI and *Asc*I sites and cloned into *pW25* to make XGL235. This fragment contains the *yellow* transcription unit and the body enhancer, but lacks the wing enhancer. Second, PCR primers containing *Not*I sites generated a 3 kb fragment (y+5234 to y+8184 relative to the *y*TSS) to make *P[y^Δ1A-2^ target]* or a 3.5 kb fragment (y+5826 to y+9318 relative to the *y*TSS) to make *P[y^Δ1A-2/Δ1A-2′^ target]*. In all cases, PCR fragments were sequenced to confirm appropriate amplification. For targeting, we generated transgenic lines in a *y^1^ w^1118^* mutant background. Gene targeting followed the procedure outlined in [59]. A combination of Southern and PCR analyses identified correctly targeted events. To remove the *w^hs^* gene, red-eyed males carrying a targeted deletion event were crossed to females carrying Cre recombinase, as described in [62]. The white-eyed flies were collected and used to establish homozygous stocks. Deletion events were confirmed by PCR amplification and sequence analyses.

### Rapid Amplification of cDNA Ends (RACE)

The structures of the *yar* RNAs were determined using RACE of total RNA isolated from 6–12 hour CS embryos. In the 3′-RACE experiments, 5 µg of RNA were reverse transcribed using the adaptor oligo-dT primer (3′-RACE kit, Invitrogen), and cDNA was amplified using a *yar* specific primer (1 µM) and the abridged universal primer (80 nM, Invitrogen). Several products were identified by agarose gel electrophoresis, gel purified and cloned into the TOPO vector (Invitrogen). Sequencing and BLAST search identified three *yar* splice variants that shared a common distal exon and poly-A signal. In the 5′-RACE experiments, 5 µg of RNA were reverse transcribed with a *yar* specific primer (100 nM), purified over a S.N.A.P column (Invitrogen) to remove unincorporated nucleotides and primers, and C-tailed at 4° for 2 hours, using terminal deoxynucleotidyl transferase. Tailed cDNAs were amplified with nested *yar* specific primers (400 nM) and an abridged anchor primer (400 nM, Invitrogen). PCR products were directly cloned into the TOPO vector. Forty-eight clones were analyzed by restriction digestion, revealing nine classes of insert. At least one representative of each class was sequenced. BLAST analyses of these data identified ten alternative splice variants and three alternative start sites. Both the 3′-RACE and 5′-RACE were performed on two independent RNA isolations. Gene-specific primer sequences are available upon request.

### Northern and Real-Time PCR Analyses

RNA was isolated from staged embryos collected from cages of wild type (CS) flies, using the NaDodSO_4_/phenol technique [63]. Five µg of oligo-dT selected polyA^+^ RNA was used in northern analyses and hybridized with radiolabeled fragments corresponding to *y* (a *Cla*I-*Bgl*II fragment, representing +2466 to +4815 relative to the *y*TSS), *yar* (EST DN154052, 418 bp ) and *ac* (a PCR fragment representing +115 to +531 relative to the *ac*TSS). Hybridization with sequences corresponding to the ribosomal gene, *RpL32*, served as a loading control. For real-time PCR experiments, RNA was isolated from embryos and pupae from three lines: CS, *y^Δ1A-2^* line XGL339-23-38, *y^Δ1A-2/Δ1A-2′^* line XGL426-41-4. RNA isolation and real-time PCR analyses were performed as described in [16]. PCR primers amplified 100–200 bp fragments. *y* primers flanked the intron. *yar* primers were in the invariant fourth exon, to ensure quantification of all transcripts. Primer sequences are available upon request. Duplicate or triplicate reactions were performed and averaged, with the difference among the replicates no greater than 0.5 cycle threshold (C_T_). At least three independent experiments were performed for each primer set from two independent RNA samples. The expression level of each gene was determined using *Ras64B* as an internal control (ΔC_T_). The fold change in expression of each gene relative to the wild type (CS) value was determined with the ΔΔC_T_ method.

## Supporting Information

Figure S1 Southern analysis of *y-ac* locus in homologous recombinant lines. Genomic DNA was isolated from ten flies, digested with *Eco*RV (NEB) and run on a 1% agarose gel. Flies analyzed were the parental *y^1^w^1118^* line, the *P[yΔ1A-2 target]* or *P[yΔ1A-2/1A-2′]* transgenic (TG) lines, homologous recombinants carrying the *w^hs^* gene (*y^Δ1A-2w^* and *y^Δ1A-2/1A-2′w^*), and homologous recombinants deleted for *w^hs^* gene (*y^Δ1A-2^* and *y^Δ1A-2/1A-2′^*). DNAs were transferred to Nytran and hybridized with a ^32^P-labeled probe made with *Cla*I to *Bgl*II fragment of *y* gene (black bar, Figure 5). The probe recognizes an endogenous band of 7.6 kb in *y^1^w^1118^* flies, and transgene band of 4.5 kb. Correct recombination events removed the endogenous band. Excision of *w^hs^* gene with Cre recombinase lead to appearance of a new *Eco*RV site at the *LoxP* element in *y^Δ1A-2^* line (3 kb band). A similar event did not occur in the *y^Δ1A-2/1A-2′^* line, therefore a smaller band is seen due to the ∼1.0 kb deletion of the Su(Hw) BSs (6.7 kb band).
(5.15 MB TIF)

Figure S2 Definition of parameters for semi-quantitative PCR analyses. Indicated volumes of cDNA were used as a template for amplification by the *ac*, *yar*, *y* and *Ras64B* primers for the number of cycles shown at the right. Ethidium-stained PCR products from each input were analyzed. These studies demonstrated that at the cycle number shown, each primer set produced an increasing amount of product with increasing input. In the semi-quantitative PCR reactions shown in Figure 6, 1 µl of template was used for the given number of cycles.
(2.17 MB TIF)

Figure S3 Analysis of RNA accumulation from 1A region genes in wild type and mutant backgrounds. Quantitative real time PCR (Q-PCR) was used to determine levels of *y*, *ac* and *sc* mRNA accumulation from RNAs isolated during development from wild type and mutant lines. Individual transcript levels defined by Q-PCR were normalized to *Ras64B* for amount of input cDNA (ΔC_T_). A larger ΔC_T_ indicates a reduction in RNA. Error bars indicate standard deviation of values obtained from analyses of three independently isolated RNAs. No significant changes in RNA accumulation relative to wild type were detected.
(10.6 MB TIF)
