# Supplementary figures and images for: Context Differences Reveal Insulator and Activator Functions of a Su(Hw) Binding Region
Source: PLoS Genet. 2008 Aug 15;4(8):e1000159. doi: 10.1371/journal.pgen.1000159 (PMC2493044; doi:10.1371/journal.pgen.1000159)

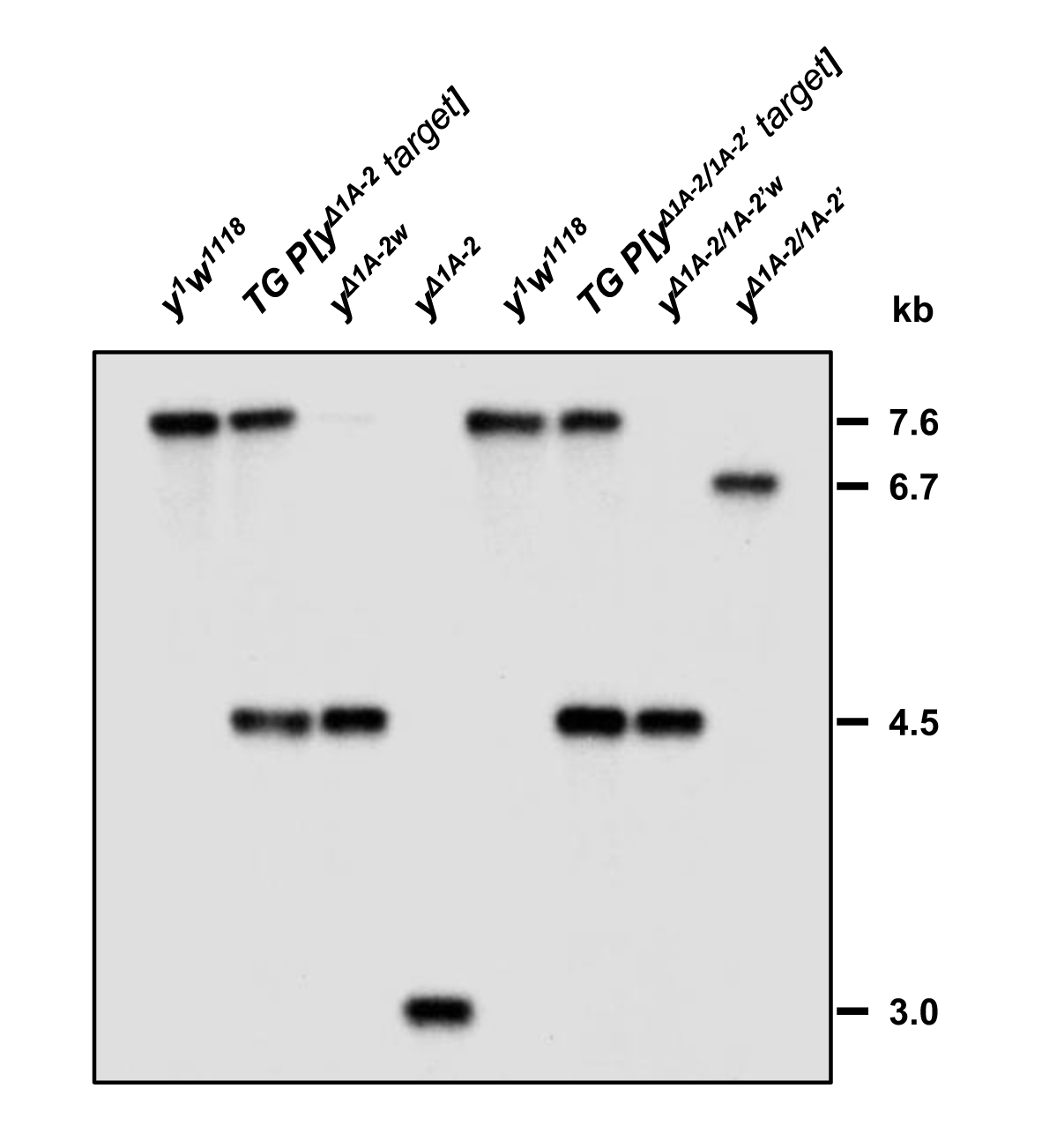

Supplement: Figure S1 — Southern analysis of y-ac locus in homologous recombinant lines. Genomic DNA was isolated from ten flies, digested with EcoRV (NEB) and run on a 1% agarose gel. Flies analyzed were the parental y1w1118 line, the P[yΔ1A-2 target] or P[yΔ1A-2/1A-2′] transgenic (TG) lines, homologous recombinants carrying the whs gene (yΔ1A-2w and yΔ1A-2/1A-2′w), and homologous recombinants deleted for whs gene (yΔ1A-2 and yΔ1A-2/1A-2′). DNAs were transferred to Nytran and hybridized with a 32P-labeled probe made with ClaI to BglII fragment of y gene (black bar, Figure 5). The probe recognizes an endogenous band of 7.6 kb in y1w1118 flies, and transgene band of 4.5 kb. Correct recombination events removed the endogenous band. Excision of whs gene with Cre recombinase lead to appearance of a new EcoRV site at the LoxP element in yΔ1A-2 line (3 kb band). A similar event did not occur in the yΔ1A-2/1A-2′ line, therefore a smaller band is seen due to the ∼1.0 kb deletion of the Su(Hw) BSs (6.7 kb band). (5.15 MB TIF) [file pgen.1000159.s001.tif]

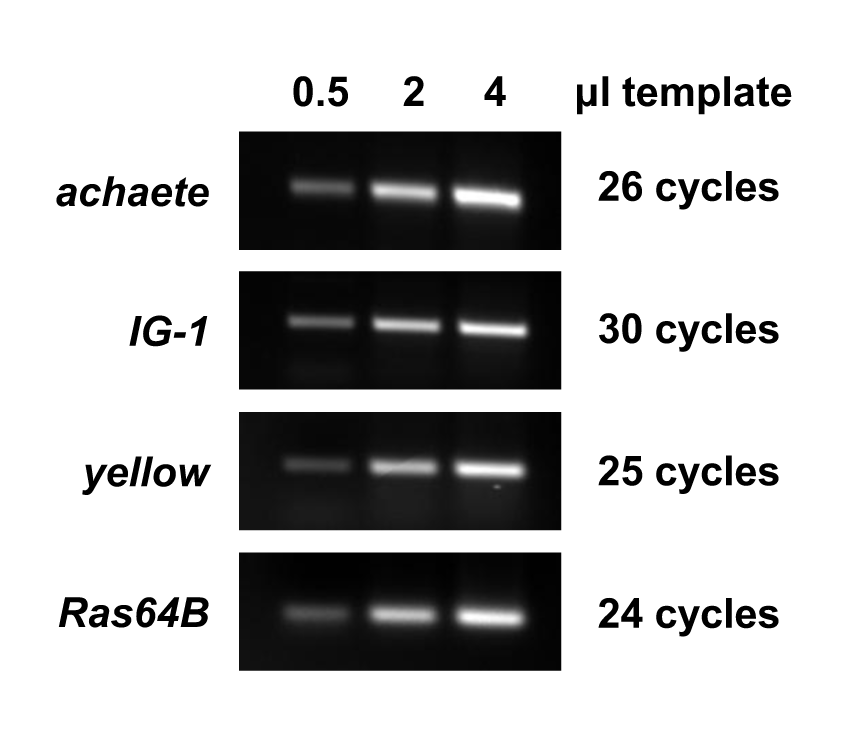

Supplement: Figure S2 — Definition of parameters for semi-quantitative PCR analyses. Indicated volumes of cDNA were used as a template for amplification by the ac, yar, y and Ras64B primers for the number of cycles shown at the right. Ethidium-stained PCR products from each input were analyzed. These studies demonstrated that at the cycle number shown, each primer set produced an increasing amount of product with increasing input. In the semi-quantitative PCR reactions shown in Figure 6, 1 µl of template was used for the given number of cycles. (2.17 MB TIF) [file pgen.1000159.s002.tif]

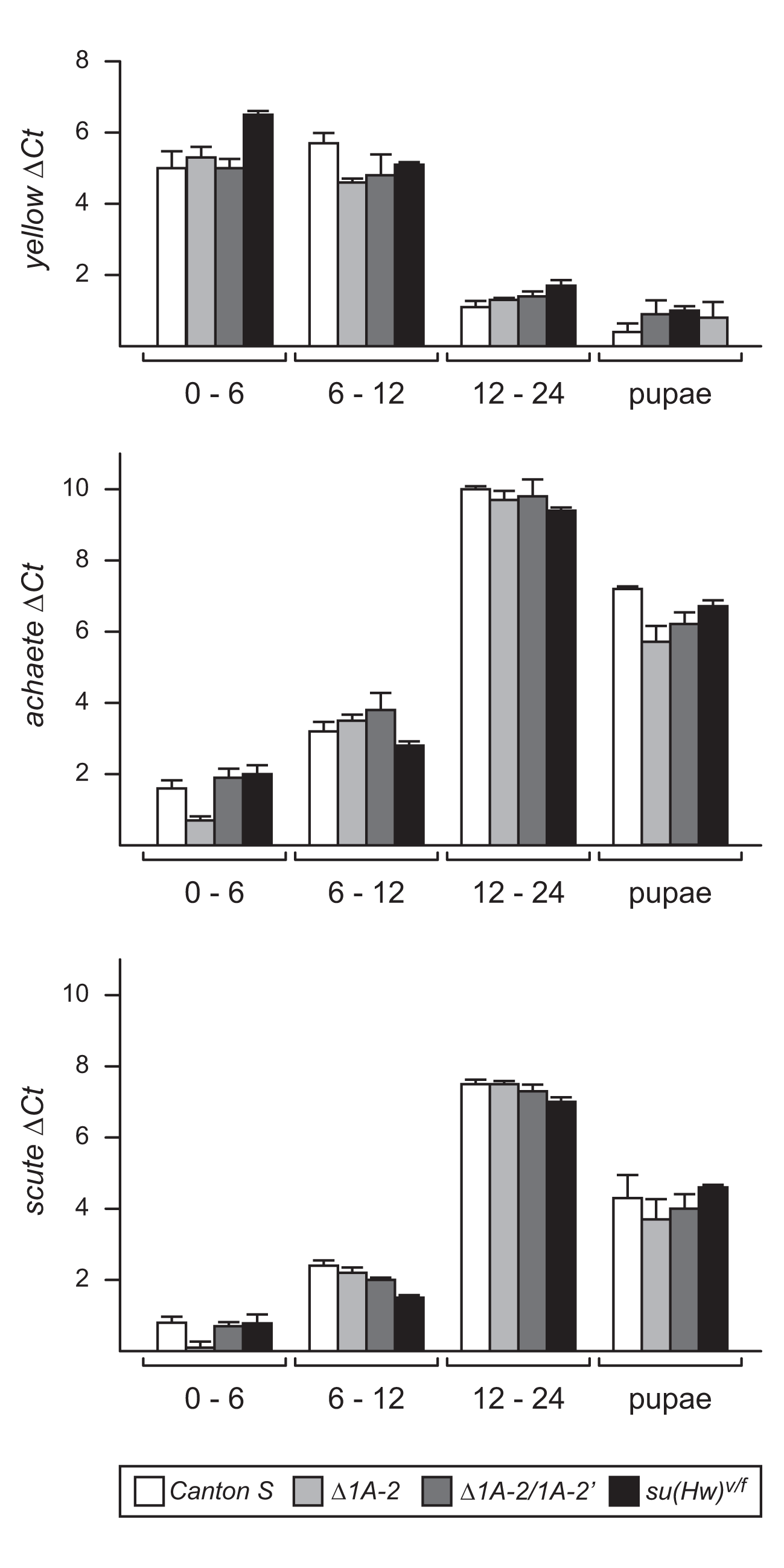

Supplement: Figure S3 — Analysis of RNA accumulation from 1A region genes in wild type and mutant backgrounds. Quantitative real time PCR (Q-PCR) was used to determine levels of y, ac and sc mRNA accumulation from RNAs isolated during development from wild type and mutant lines. Individual transcript levels defined by Q-PCR were normalized to Ras64B for amount of input cDNA (ΔCT). A larger ΔCT indicates a reduction in RNA. Error bars indicate standard deviation of values obtained from analyses of three independently isolated RNAs. No significant changes in RNA accumulation relative to wild type were detected. (10.6 MB TIF) [file pgen.1000159.s003.tif]
